# 

**Published:** 1843-09-30

**Authors:** 


CONTENTS.

A Lecture on Sanguineous Tumours, delivered at the Hotel Dieu, Paris, May 23d, 1843. By M. Roux, - - - 217

*Bibliographical Notices.*

Principles of Human Physiology, with their application to Pathology, Hygiene and Forensic Medicine. By William B. Carpenter, M. D., &c. With Notes and Additions, by Meredith Clymer, M. D., &c., - - - - - - 221

Medical History of the Expedition to the Niger. By James Ormiston McWilliam, M. D., Senior Medical Officer of the Expedition, - - - - - - 222

Some account of the African Remittent Fever. By Morris Pritchett, M. D., - 222

Elements of Chemistry, including the applications of the Science to the arts. By Thomas Graham, F. R. S. L., &c. With Notes and Additions. By Robert Bridges, M. D., &c., - - - - 224

*Foreign Correspondence,* - - - - 225

*Retrospect of the Medical Sciences.*

Gallic Acid in Menorrhagia, - - - 226

Morbid State of the Function of the Skin in Insanity, - - - - - 227

Unhealthiness of Liverpool and its causes, 227

Antagonism of Phthisis and Intermittent Fever, - - - - - - - 227

Exhalation of Carbonic Acid from the body, 228

Population of Belgium, - - - - 228

M. Peligot on the Chemical Composition of Tea, - - - - - - - 228

Spurious Preparations of Mercury, - - 228

